# Sorting nexin-1 is a candidate tumor suppressor and potential prognostic marker in gastric cancer

**DOI:** 10.7717/peerj.4829

**Published:** 2018-05-29

**Authors:** Xiao-Yong Zhan, Yaqiong Zhang, Ertao Zhai, Qing-Yi Zhu, Yulong He

**Affiliations:** 1 The First Affiliated Hospital, Sun Yat-sen University, Guangzhou, China; 2 Guangzhou KingMed Center for Clinical Laboratory, Guangzhou, China; 3 KingMed School of Laboratory Medicine, Guangzhou Medical University, Guangzhou, China

**Keywords:** Gastric cancer, Sorting nexin-1, Tumor suppressor, Prognosis

## Abstract

Sorting nexin-1 (SNX1) is an important functional protein in cell endocytosis, efflux, protein sorting, cell signal transduction, etc; however, the expression, the role and clinical relevance of SNX1 have not been investigated in gastric cancer (GC). In this study, we first performed a bioinformatics investigation using the data obtained from The Cancer Genome Atlas (TCGA) database. The result showed that SNX1 mRNA levels were significantly lower in GC tissues than in paracancerous tissues. In a study of 150 cases of GC, including 60 cases with paired paracancerous and cancer tissues and 90 cases with detailed follow-up information, SNX1 expression was analyzed by immunohistochemistry. Our study on paired paracancerous and cancer tissues showed that SNX1 protein expression remarkably decreased in GC tissues (50/60, 83.33%). A study on 90 patients with detailed follow-up information showed that tumors with higher SNX1 protein level were correlated with better clinicopathologic stages (*p* = 0.0285), nodal status (*p* = 0.0286), smaller tumor sizes (*p* = 0.0294) and a better survival rate in patients with GC (*p* = 0.0245). Univariate analysis of the 90 patients with GC showed that low-level SNX1 was significantly correlated with decreased overall survival of GC patients (*p* = 0.008), and associated with a relatively higher cumulative hazard of death. Exogenous expression of SNX1 inhibited the growth, migration, invasion and promoted the apoptosis and enhanced the sensitivity of GC cells to the chemotherapeutic drug 5-Fluorouracil (5-Fu) in vitro, while knockdown of SNX1 by short hairpin RNA (shRNA) significantly promoted the growth, migration, invasion and reduced the apoptosis and the sensitivity of GC cells to 5-Fu. SNX1 also showed to influence the levels of epithelial-mesenchymal transition markers including Vimentin, Snail, and E-cadherin in GC cells in vitro. Taken together, we propose here that SNX1 serves as a tumor suppressor and prognostic marker that reduces tumor cell malignancy for GC.

## Introduction

Gastric cancer (GC) is the fifth most common malignancy worldwide and causes the third most cancer-related death (Ferlay et al., 2015). There are many deaths caused by GC in China every year which account for nearly half of the world’s GC deaths (Sheets, 2014). Several risk factors have been identified for GC, including *Helicobacter pylori* infection, *Epstein-Barr* virus infection, and dietary factors (Carrasco & Corvalan, 2013; Chen et al., 2015; Guggenheim & Shah, 2013). Mounting evidence showed that genetic factors, abnormal signal transduction and their downstream effects on cellular processes played important roles in GC (Gigek et al., 2017; McLean & El-Omar, 2014; Razzak, 2014). The pathogenesis of GC is still quite complex although numerous studies investigated molecular mechanisms of the initiation and development of GC. Therefore, investigating the pathogenesis of GC, finding new biomarkers with higher sensitivity and representability are desired.

Sorting nexin-1 (SNX1), the first member of the sorting nexins (SNXs) found in mammals, was discovered in 1996 by a yeast two-hybrid experiment, in which SNX1 could interact with epidermal growth factor receptor (EGFR) and accelerated its degradation (Kurten, Cadena & Gill, 1996). Further works demonstrated that SNX1 played an important role in cell endocytosis, efflux, protein sorting, cell signal transduction, membrane transport and remodeling, and organelle movement (Bryant et al., 2007; Gokool, Tattersall & Seaman, 2007; Nishimura et al., 2011; Zhang et al., 2013). SNX1 is associated with many intracellular signaling pathways. Downregulation of SNX1 promotes HGF-induced MET (product of oncogene *c-met*) endocytosis and leads to the phosphorylation of MET in human lung cancer cell line, and then activates RTK/RAS signaling pathway and leads to the activation of cell proliferation and inhibition of cell apoptosis, and increase cell malignancy (Nishimura et al., 2015; Peschard & Park, 2007; Stuart et al., 2000). SNX1 is also reported to suppress EGFR membrane recycling and activate EGFR/PI3K/AKT signaling pathway in lung cancer cells (Nishimura et al., 2015). Besides above, SNX1 is associated with some other pathologies including bacterial infection and hypertension. SNX1 defines an early phase of Salmonella-containing vacuole-remodeling during Salmonella infection thus it could regulate intracellular bacterial progression and replication (Bujny et al., 2008). SNX1 is required for dopamine D5 receptor function in human renal proximal tubule cells (Villar et al., 2010), and its loss results in D5 dopamine receptor dysfunction in human renal proximal tubule cells, thus it may be associated with hypertension (Villar et al. , 2013). SNX1 is also reported to have an association with the development and metastasis of some tumors, including lung cancer and colon cancer (Bian et al., 2016; Chen et al., 2014; Holdren, Her & Parks, 2005; Nguyen et al., 2006; Nishimura et al., 2012). Despite these findings, the expression of SNX1 in other tumor tissues and the role of SNX1 in the development and metastasis of other tumor tissues remains unknown and its detailed clinical value remains to be elucidated. Thus, in this study, we investigated the SNX1 expression, the potential functional mechanism, and its prospect as a potential prognostic marker in GC.

## Materials and Methods

### The Cancer Genome Atlas (TCGA) database analysis

To determine the potential role of SNX1 in GC, we performed a bioinformatics analysis using TCGA database (Tomczak, Czerwinska & Wiznerowicz, 2015) (https://cancergenome.nih.gov/). Stomach adenocarcinoma gene expression data were manually retrieved from the UCSC Cancer Genomics Browser (https://genome-cancer.ucsc.edu) and then extracted and checked. Data processing followed the instruction of the database and report elsewhere (Wang, Jensen & Zenklusen, 2016). SNX1 mRNA expression levels in 384 GC tissues and 37 non-cancerous paracancerous tissues (NCTs) were obtained and analyzed (Table S1).

### Tissue specimens and immunohistochemical staining

Tumor specimens were obtained from 150 patients with primary GC and underwent a gastrectomy at the First Affiliated Hospital, Sun Yat-sen University from 2004 to 2016. Among them, sixty pairs of human GC tissues and adjacent NCTs were collected between 2009 and 2016. These tissues were used to research differential expression of SNX1 protein between tumor tissues and NCTs. Another 90 GC tissue samples were collected between 2004 to 2007. Detailed follow-up information was included in the patients with these tissues. Follow-up study was conducted up to December 31, 2013. They were used for the study of correlation of SNX1 with clinicopathologic characteristics and for the survival analysis and Cox Regression analysis. All human materials were obtained with informed consent, and this project was approved by the ICE for Clinical Research and Animal Trials of the First Affiliated Hospital, Sun Yat-sen University (approval ID 2017226). We retrieved the information, including gender, age, stage of disease and histopathological factors from the patient hospital charts. Patients’ data and clinical information are summarized in Table S2. The pathologic features of the specimens were classified based on the eighth edition of the tumor-node-metastasis (TNM) classification of the Union for International Cancer Control (UICC) and the American Joint Committee on Cancer (AJCC). Both tumor and adjacent NCTs were histologically examined. Tissue microarray construction was performed as described elsewhere (Fedor & De Marzo, 2005). The primary antibody was rabbit monoclonal antibody against SNX1 (1:100; Abcam). Quantifiable levels of SNX1 in the tissues were measured by histochemistry score (H-score), which was obtained based on the proportion and intensity of brown staining cells with calculation formula: H-score = (% of cells stained at intensity 1 × 1) + (% of cells stained at intensity 2 × 2) + (% of cells stained at intensity 3 × 3) (Yeo et al., 2015).

### Cell lines and culture

Four human gastric cell lines, including a non-cancerous gastric epithelium cell line, GES-1 (Ke, Ning & Wang, 1994), and three GC cell lines, MKN-1, SGC-7901 and BGC-823, were purchased from the Type Culture Collection of the Chinese Academy of Sciences. All cells were maintained in RPMI 1640 medium supplemented with 10% fetal bovine serum (FBS). Cells were grown under a 5% CO2 atmosphere at 37 °C.

### RNA extraction and quantitative real-time polymerase chain reaction (qRT-PCR) analysis

Total RNA was extracted by Trizol (Invitrogen, Carlsbad, CA, USA) according to the manufacturer’s instructions. qRT-PCR was performed using TransScript Green One-Step qRT-PCR SurperMix (Transgene, Shenzen, China), in a total volume of 20 µl containing RNA, 2 ×SurperMix, and 0.2 µM of each primer. The primers used for qRT-PCR were: SNX1-F, 5′-GTGGTGCTGGTCTCCTCAAG-3′; SNX1-R, 5′-CGCTGCTCCTCACACTCTAC-3′. GAPDH-F, 5′-GCACCGTCAAGGCTGAGAA-3′; GAPDH-R, 5′-GTGAAGACGCCAGTGGACTC-3′.

### Vector construction and transfection

For SNX1 exogenous expression vector construction, the SNX1 coding region was amplified from the cDNA of GES-1 cells by using the following primers: 5′-GCCACCATGGCGTCGGGTGGTGGT-3′ and 5′-GGAGATGGCCTTTGCCTCAGGA-3′. The PCR product was then subcloned into the pEASY-Blunt M3 plasmid (Transgene, Beijing) to generate SNX1 overexpression plasmid (pEASY-SNX1). For SNX1 short hairpin RNA (shRNA) vector construction, the specific sequence used for the depletion of SNX1 (5′-CACCGTAGTGACTTTCTGGGTCTTTATTCAAGAGA TAAAGACCCAGAAAGTCA CTACTTTTTTG-3′) was synthesized and then subcloned into the pGPU6-RFP-Neo plasmid (Genepharma, Shanghai, China) to generate SNX1 downregulation plasmid (shRNA-SNX1). The product of the small interfering RNA (siRNA) of this sequence was shown to have a knockdown level of 97% for SNX1 based on Sigma MISSION^®^ shRNA. For control, pEASY-Blunt M3 empty plasmid (pEASY), and shRNA-control vector was constructed. The sequence (5′-CACCGTTCTCCGAACGTGTCACGTTTCAAGAGAACGTGACACGTTCG GAGAACTTTTTTG-3′) which generated an siRNA with no significant homology to any human gene sequences, was subcloned into the pGPU6-RFP-Neo plasmid (Genepharma, Shanghai, China) to generate shRNA-control plasmid (shRNA-control). For transient transfection assays, cells were transfected with each plasmid using Lipo6000 (Beyotime, Beijing, China), and were harvested after 48 h for further analysis.

### Western blotting (WB)

Whole-cell lysates (20 µg total protein) were immunoblotted using antibodies against SNX1 and other antibodies. The primary antibodies used in this study are summarized in Table S3. Antigen-antibody complexes were visualized by Western blotting ECL Reagent (Beyotime, Beijing, China).

### Colony formation assay and scratch wound healing assay

Transfected cells were trypsinized and counted. Five hundred cells were maintained for 15 days in RPMI1640 medium with 2% FBS, and cells were then fixed, stained and photographed.

Scratch wound healing assay was performed to determine the influence of SNX1 on cell migration. Briefly, transfected cells were counted with a cell counter (Bio-Rad). 2 ×10^6^ cells were inoculated in each well of 6-well plates for 24 h, and then a scratch wound was made by scraping the cell monolayer across the cover with a 10 µl sterile micro-pipette tips. The cells were then washed with PBS to eliminate residual floating cells. Wounded cultures were maintained in RPMI 1640 medium with 2% FBS and photographs were taken at 0 h, 12 h, 24 h, and 48 h. The migration distance of the cells was calculated by Image J (https://imagej.nih.gov/ij/).

### Transwell cultures

Transwell cultures were performed as described elsewhere (Marshall, 2011). Briefly, the cultures were set up in 24-well plates. For invasion assay, 6 ×10^4^ cells (in 300 µl RPMI1640 medium with 2% FBS) were seeded on the upper chamber of a 24-well Matrigel-coated membrane with 8 µm pores (Corning, Corning, NY, USA), and the bottom chamber was filled with RPMI1640 medium with 20% FBS. For the migration assay, 4 ×10^4^ cells (in 200 µl RPMI1640 medium with 2% FBS) cells were directly seeded on an uncoated chamber. After 48 h of culture, the membranes were fixed and then stained with 0.1% methylrosaniline chloride solution, and invasion and migration were quantified by counting five random fields under a microscope (200 ×).

### Cell proliferation and apoptosis assay

Cell proliferation was measured by a TransDetect Cell Count Kit (Transgene, Beijing, China) according to the manufacturer’s instructions. Briefly, 2,000 cells were inoculated in each well of a 96-well plate in RPMI 1,640 medium with 10% FBS. The CCK-8 solution was added to the wells at 24 h, 48 h, 72 h, 96 h, and 120 h, respectively. After two hours of incubation at 37 °C in a humidified incubator containing 5% CO2, OD450 values of these wells were obtained by using a microplate reader (SpectraMax i3; Molecular Devices, Shanghai, China). Cell apoptosis was determined by an Annexin V-FITC Apoptosis Detection Kit (Beyotime, Beijing, China), according to the manufacturer’s instruction. Because the cells transfected with shRNA-control or shRNA-SNX1 plasmid expressed red fluorescence protein, thus the propidium Iodide (PI) could not be used in the apoptosis detection. We gated the living cells (normal cells and apoptotic cells) on the basis of forward (FSC) and side (SSC) scatter characteristics, and then analysis the annexin V-FITC staining of these cells, as previously described (Zhan et al., 2014).

### Statistical analysis

Relative mRNA level of SNX1 between NCTs and GC tissues were analyzed by *t-test* or paired *t-test*. The Mann–Whitney *U* test or Kolmogorov–Smirnov test was used to analyze different H-score between variables obtained from clinicopathological parameters. Spearman’s correlation analysis was used to analyze the relationship between survival months and SNX1 H-scores. Survival curves were estimated by the Kaplan–Meier method and compared by the log-rank test. Cox Regression analysis was used to analyze the hazard ration using univariate Cox proportional hazards model. Each *in vitro* assay has at least three replications and the *t-test* was used in comparisons of variables for *in vitro* assays. *p* < 0.05 was considered significant. GraphPad Prism 6 (GraphPad Software) was used for graphing. Statistical analyses were performed using SPSS 16.

## Results

### Expression of SNX1 in GC

**Figure 1:**
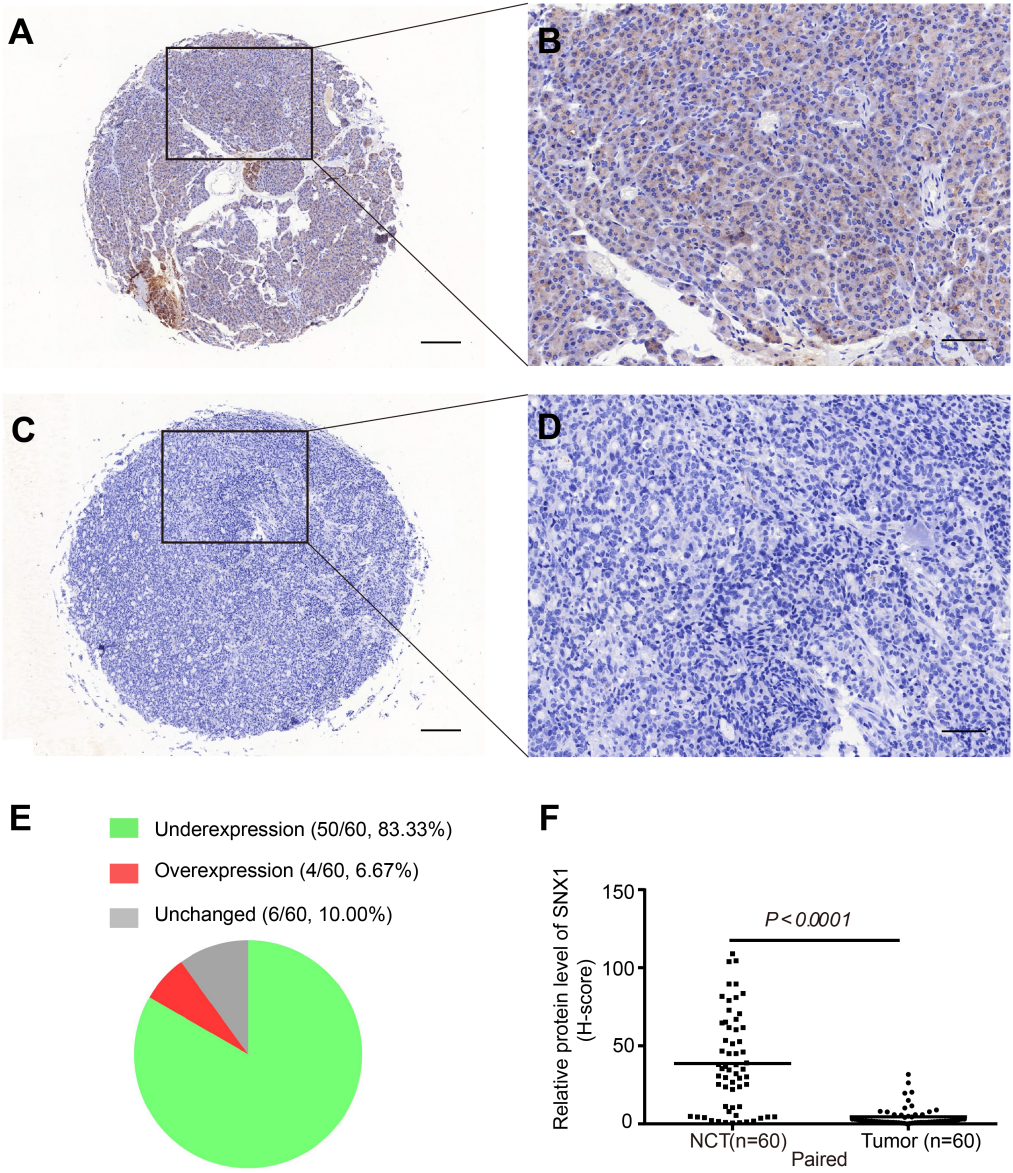
Staining for SNX1 protein expression in NCTs and GC tissues. (A–B) IHC staining of SNX1 in NCTs. (C–D) IHC staining of SNX1 in GC tissues. (E) Protein expression of SNX1 was decreased in 83.33% GC tissues by comparing the H-score of 60 paired GC tissues and adjacent NCTs. (F) Comparison between the H-scores of SNX1 in the NCTs and GC tissues. SNX1 protein expression is reduced in GC. Scale bar (A and C) 150 µm; (B and D) 50 µm.

We found differential expression of SNX1 mRNA between GC tissues and NCTs through the TCGA database analysis: the GC tissues had a lower level of SNX1 mRNA (*t-test* and paired *t-test*, *p* < 0.01, Fig. S1). Then the protein expression of SNX1 was evaluated in 60 paired GC tissues and NCTs by immunohistochemistry (IHC). As shown in Figs. 1A and  1B, SNX1 expression was exclusively in the cytoplasm of NCTs. In most cases, GC tissues presented weaker and less diffuse staining than NCTs (Figs. 1C and  1D). Overall, the protein level of SNX1 was downregulated in 83.33 % (50/60) of GC tissues compared with NCTs (Fig. 1E). We defined a more than 50% of decrease or increase of the H-score in the GC tissues compared with the paired-NCTs as underexpression or overexpression. A significant reduction of SNX1 expression was observed in the GC tissues (*p* < 0.0001, Fig. 1F). Specifically, GC tissues displayed an extremely low level of SNX1 compared with NCTs that the median H-score was 1.82 in GC tissues and 34.81 in NCTs.

### Correlation between SNX1 expression and clinicopathological parameters

To investigate the clinicopathological significance of SNX1 expression in GC, the potential associations between SNX1 expression and patients’ clinicopathological features were analyzed. Noticeably, the reduction of SNX1 protein in GC tissues was significantly associated with bigger tumor size (*p* = 0.0294), poor nodal status (*p* = 0.0286), and poor tumor p-stage (*p* = 0.0285) (Table 1). However, SNX1 expression was not associated with age, gender, histological grades and invasive depth (*p* > 0.05).

**Table 1:** SNX1 expression in human with gastric cancer.

Clinicopathological parameters	Patients, *n* (%)	H-score (Median)	^*^p values (Mann–Whitney *U* test)
**Age**			
<60	59 (66)	14.58	0.264
≥60	31 (34)	16.56	
**Gender**			
Male	54 (60)	15.56	0.602
Female	36 (40)	15.10	
**Pathology-stages (pTNM)**			
I–II	41 (46)	15.90	**0.0285**^&^
III–IV	49 (54)	13.81	
**Histological grades**			
2	22 (24)	14.39	0.902
3	68 (76)	15.72	
**Lymph node metastasis**			
N0+N1	50 (56)	16.58	**0.0286**
N1+N2	40 (44)	13.00	
**Invasive depth**			
T1+T2	16 (18)	16.23	0.989
T3+T4	73 (82)	14.60	
**Tumor size^#^**			
Small	55 (61)	20.03	**0.0294**
Big	35 (39)	12.83	

**Notes.**

# Tumor diameter ≤5 centimeters was defined small and >5 centimeters was defined big.

* *p* value <0.05 was considered significant.

& Kolmogorov–Smirnov test was used to compare the levels of SNX1 between different pTNM groups, and the *p* value was 0.0645 through Mann–Whitney U test.

### Association of SNX1 expression with the survival rate of GC patients

**Figure 2:**
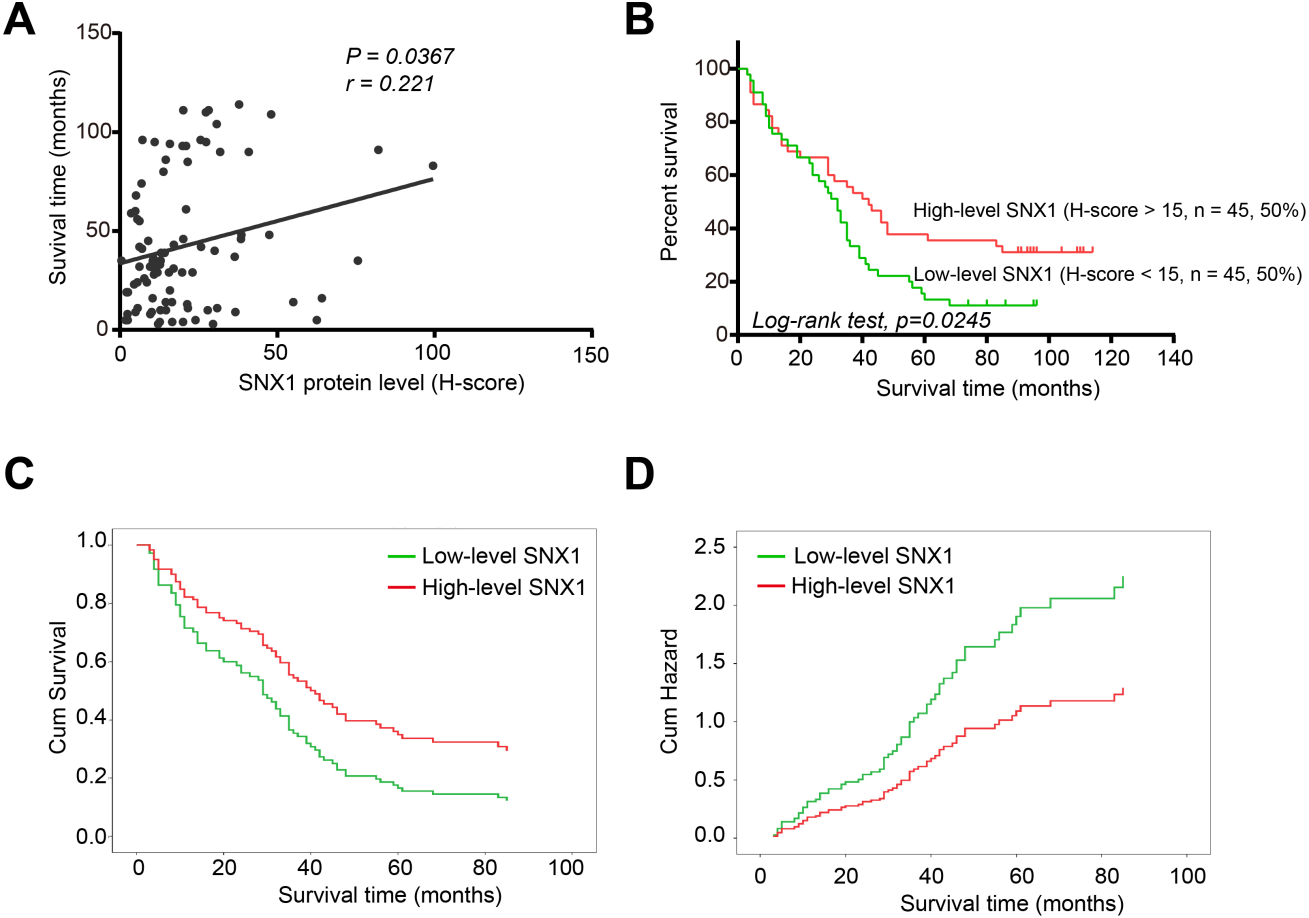
SNX1 expression is related to poor prognosis of GC patients. (A) The protein level of SNX1 was positively correlated with survival time in the 90 patients. (B) Kaplan-Meier survival curve and log-rank test were used to evaluate the overall survival based on the protein levels of SNX1. (C) Survival curve based on the Cox Regression analysis. High-level SNX1 was defined as H-score >15, while low-level SNX1 was defined as H-score <15 and the 90 patients were divided into high-level and low-level groups (each group had 45 patients). (D) Cumulative hazard of death associated with low-level SNX1 or high-level SNX1.

SNX1 protein levels were positively correlated with overall survival months of the patients (Fig. 2A). A higher survival rate was observed in patients with high-level SNX1, and the 5-year survival rate in patients with high-level SNX1 was much higher than those with low-level SNX1 (35.56% for the patients with high-level SNX1 and 13.33% for the patients with low-level SNX1; Fig. 2B). Collectively, these results suggest that the downregulation of SNX1 is involved in the development, progression, and prognosis of GC and SNX1 may be a potential prognostic marker of GC. Univariate analysis also showed that the low-level SNX1 was correlated with decreased overall survival of GC patients (Table 2). We brought the clinicopathological features that thought may be associated with survival including the pathology-stages, nodal status, and tumor histological grades into the multivariate analysis. Low-level SNX1 was also shown to be an independent risk factor for survival. Patients with low-level SNX1 have a high risk of death (hazard ration = 1.708, 95 % CI [1.060–2.751], *p* = 0.028). The survival curve based on Cox Regression analysis was similar to that based on Kaplan–Meier method (Fig. 2C), and the patients with low-level SNX1 also had a higher cumulative hazard of death than those with high-level SNX1 (Fig. 2D).

**Table 2:** Univariate Cox’s analysis of overall survival for the 90 patients with GC.

**Parameters**	*p* values	Hazard ration	95% CI
Age (<60/≥60)	0.402	1.237	0.752–2.037
Gender (male/female)	0.122	0.672	0.406–1.113
pTNM (I–II/III–IV)	0.982	1.009	0.459–2.221
Histological grades (2/3)	0.257	0.715	0.400–1.277
Lymph node metastasis (N0-N1/N2-N3)	0.273	0.670	0.327–1.372
Invasive depth (T1+T2/T3+T4)	0.841	0.926	0.438–1.958
Tumor size (≤5/>5 centimeters)	0.790	0.932	0.554–1.568
SNX1 level (low/high)	**0.008**	2.141	1.220–3.759

### Effects of SNX1 on GC cell behavior *in vitro*

**Figure 3:**
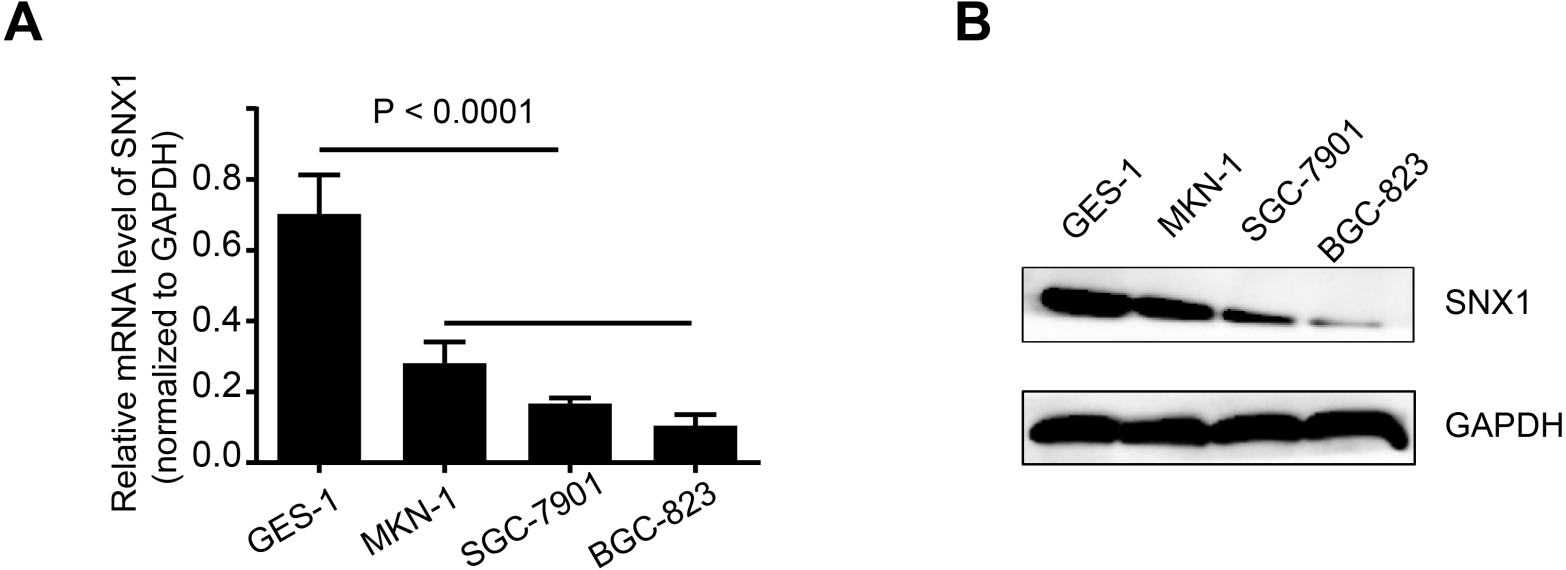
SNX1 expression in gastric cell lines. (A) Relative mRNA levels (normalized to GAPDH) of SNX1 between non-cancerous gastric epithelium cell line and GC cell lines. (B) Detection of SNX1 protein expression by WB.

To further investigate the role of SNX1 in the GC cells, first, we performed qRT-PCR assays to quantify SNX1 expression in four cell lines. As shown in Fig. 3A, qRT-PCR results revealed that GC cell lines including MKN-1, SGC-7901 and BGC-823 expressed relative lower mRNA level of SNX1 compared with the normal human gastric mucosal epithelium GES-1 cell line. WB results also showed that SNX1 was weakly detected in the GC cell lines (Fig. 3B).

For a functional investigation of SNX1, the GC cell lines with a moderate level of SNX1 (MKN-1 and SGC-7901) were selected for exogenous SNX1 expression or knockdown of SNX1 by shRNA and further analysis. We monitored the effect of SNX1 on the capacity of GC cell colony formation, cell migration and invasion, cell proliferation, cell apoptosis and cell sensitivity to traditional chemotherapeutic drugs 5-Fluorouracil (5-Fu) and oxaliplatin.

**Figure 4:**
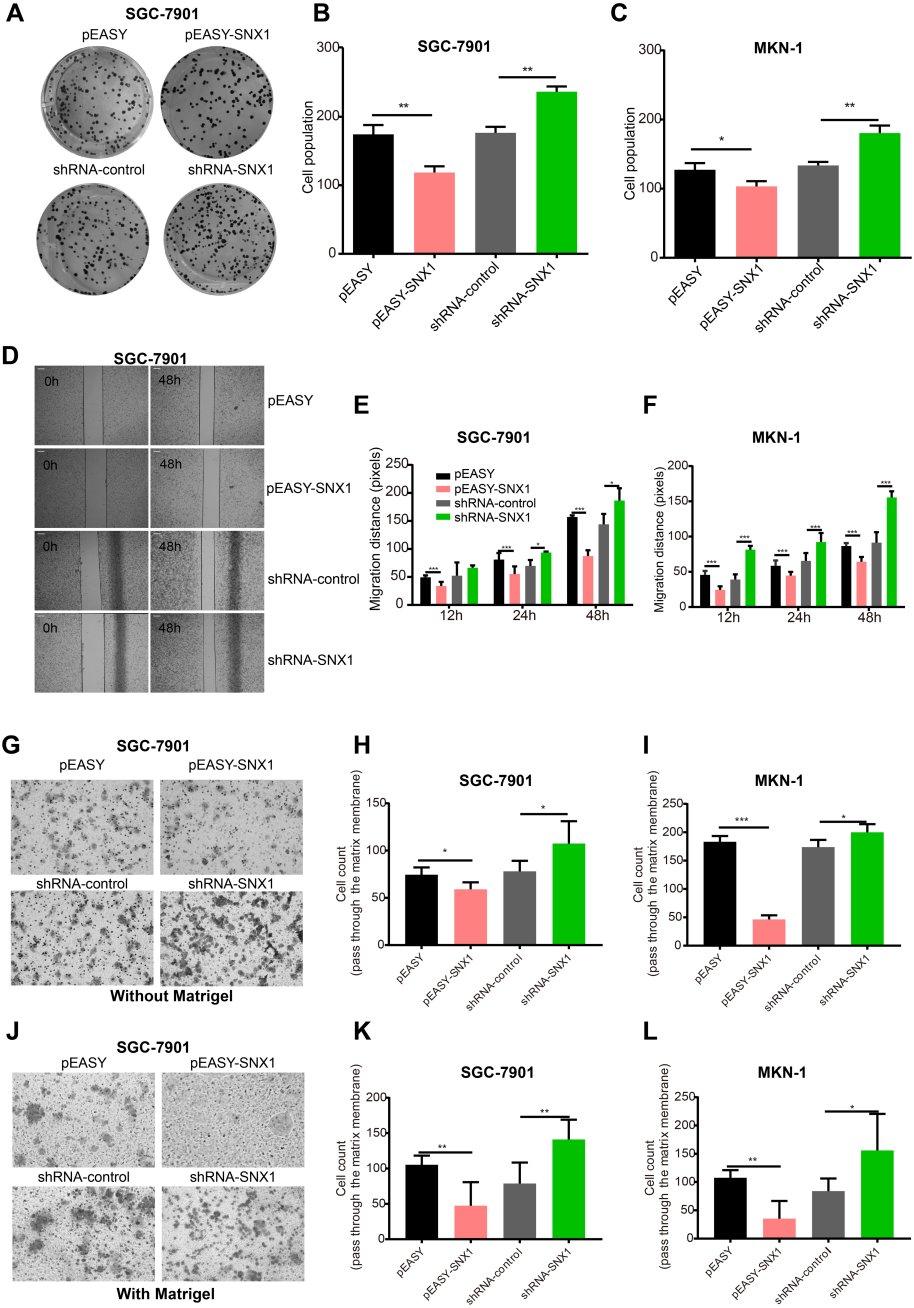
SNX1 inhibits the colony formation, the migration and invasion of GC cell *in vitro*. (A) Representative photographs of colony formation of GC cells. (B–C) Overexpression of SNX1 in GC cells led to the inhibition of the ability of colony formation while knockdown of SNX1 led to the promotion of colony formation in GC cells. (D) Representative photographs of scratch wound healing assay results. (E–F) Scratch wound healing assay showed that SNX1 inhibited the migration of GC cells while down-regulation of SNX1 promoted GC cell migration. (G) Representative photographs of transwell migration assay. (H–I) Transwell migration assay showed that SNX1 inhibited the migration of GC cells while downregulation of SNX1 promoted GC cell migration. (J) Representative photographs of matrigel invasion assay. (K–L) Matrigel invasion assay showed that SNX1 inhibited the invasion ability of GC cells while downregulation of SNX1 promoted GC cell invasion. *p < 0.05; **p < 0.01; ***p < 0.001. Scale bar (B) 200 µm.

As shown in Fig. S2, the transfection of pEASY-SNX1 or shRNA-SNX1 could upregulate or downregulate SNX1 expression. Overexpression of SNX1 inhibited colony formation of GC cells, while knockdown of SNX1 enhanced the colony formation ability (Figs. 4A– 4C). To determine whether SNX1 may play a role in cell migration, we performed a scratch wound healing assay and a transwell migration assay. Our results showed that overexpression of SNX1 inhibited the migration ability, while knockdown of SNX1 enhanced the migration ability (Figs. 4D– 4I). Matrigel invasion assay showed that overexpression of SNX1 inhibited the invasion ability while knockdown of SNX1 enhanced the invasion ability of GC cells (Figs. 4J– 4L). These result suggested that SNX1 inhibits the invasion and metastasis abilities of GC cells.

**Figure 5:**
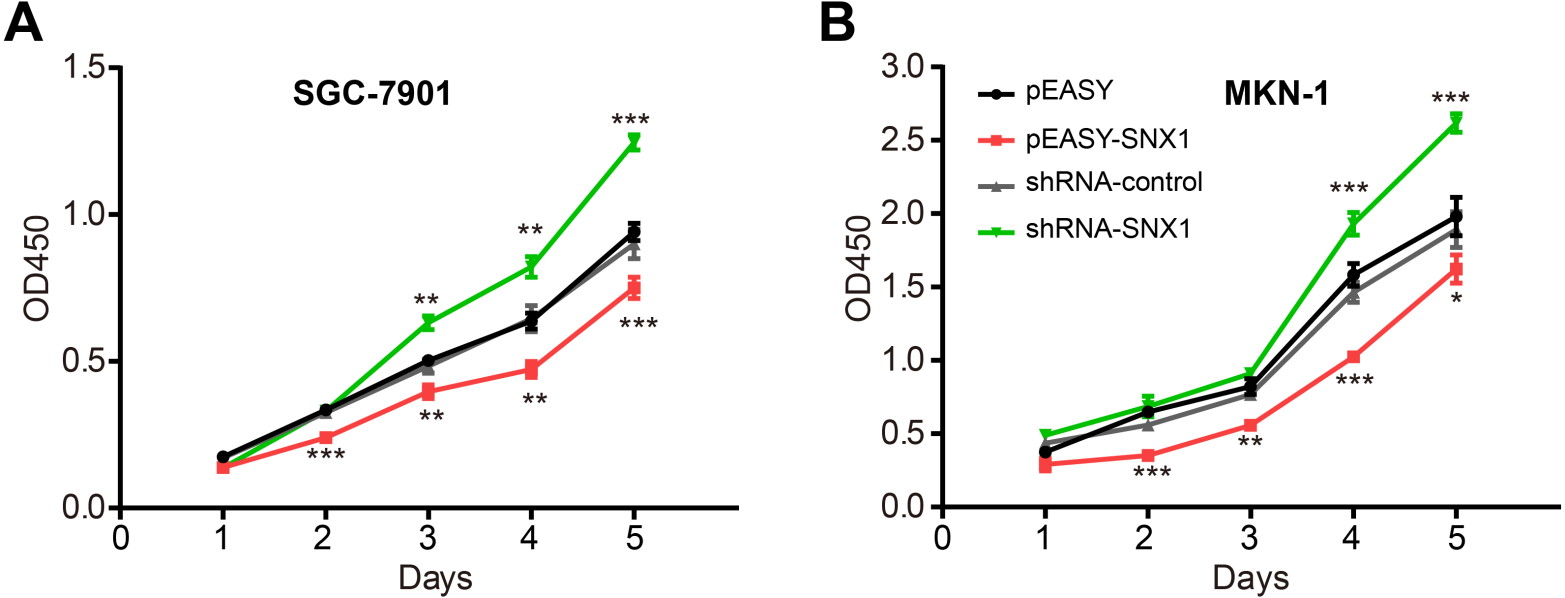
SNX1 inhibits the proliferation of GC cells *in vitro*. CCK-8 assay showed that SNX1 overexpression repressed the proliferation in (A) SGC-7901 cells and (B) MKN-1 cells while SNX1 downregulation enhanced the cell proliferation.

**Figure 6:**
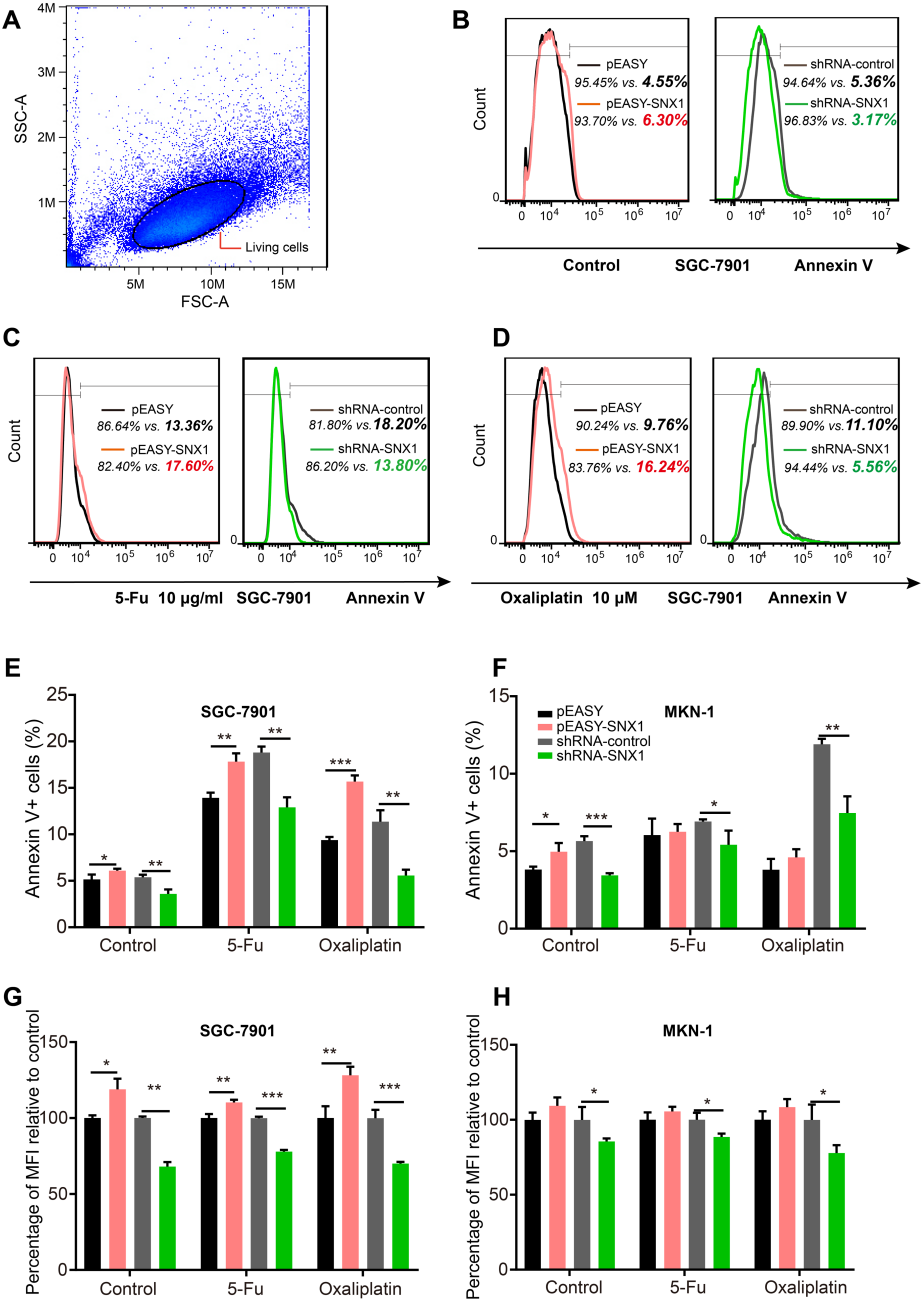
Flow cytometry assays to detect apoptotic levels of GC cells treated with 5-Fu or oxaliplatin. (A) Living cells (normal cells and apoptotic cells) gated on the basis of FSC and SSC scatter characteristics. (B–D) The figures present the difference in the apoptotic levels in the different GC cells (transfected with different vectors) treated without or with 5-Fu/oxaliplatin. The apoptotic levels of the gated cells were analyzed by both measuring the percentage of apoptotic cells and the MFI of these cells. Typical percentages of normal/apoptotic cells are shown in the histogram charts. Flow cytometry histograms showed that knockdown of SNX1 reduced the percentage of annexin V+ GC cells treated with 5-Fu or oxaliplatin, which meant the apoptosis inhibition of GC cells in vitro; while overexpression of SNX1 increased the percentage of annexin V+ GC cells treated with 5-Fu oxaliplatin, which meant the promotion GC cells apoptosis promotion of GC cells. The overlapping flow cytometry histograms also showed that knockdown of SNX1 reduced the apoptotic MFI of GC cells treated with 5-Fu oxaliplatin while overexpression of SNX1 increased the apoptotic MFI of these cells. (E–F) Statistical analysis showed that knockdown of SNX1 significantly reduced the percentage of annexin V+ GC cells treated with 5-Fu or oxaliplatin. (G–H) Statistical analysis showed that knockdown of SNX1 significantly reduced the annexin V-FITC MFI of GC cells. The MFI of GC cells transfected with pEASY-SNX1 or shRNA-SNX1 was normalized to control (the control is pEASY for pEASY-SNX1, and is shRNA-control for shRNA-SNX1). The bold italic numbers in the flow cytometry histograms indicate percentage of annexin V+ cells in the cells with different treatment. *p < 0.05; **p < 0.01; ***p < 0.001.

**Figure 7:**
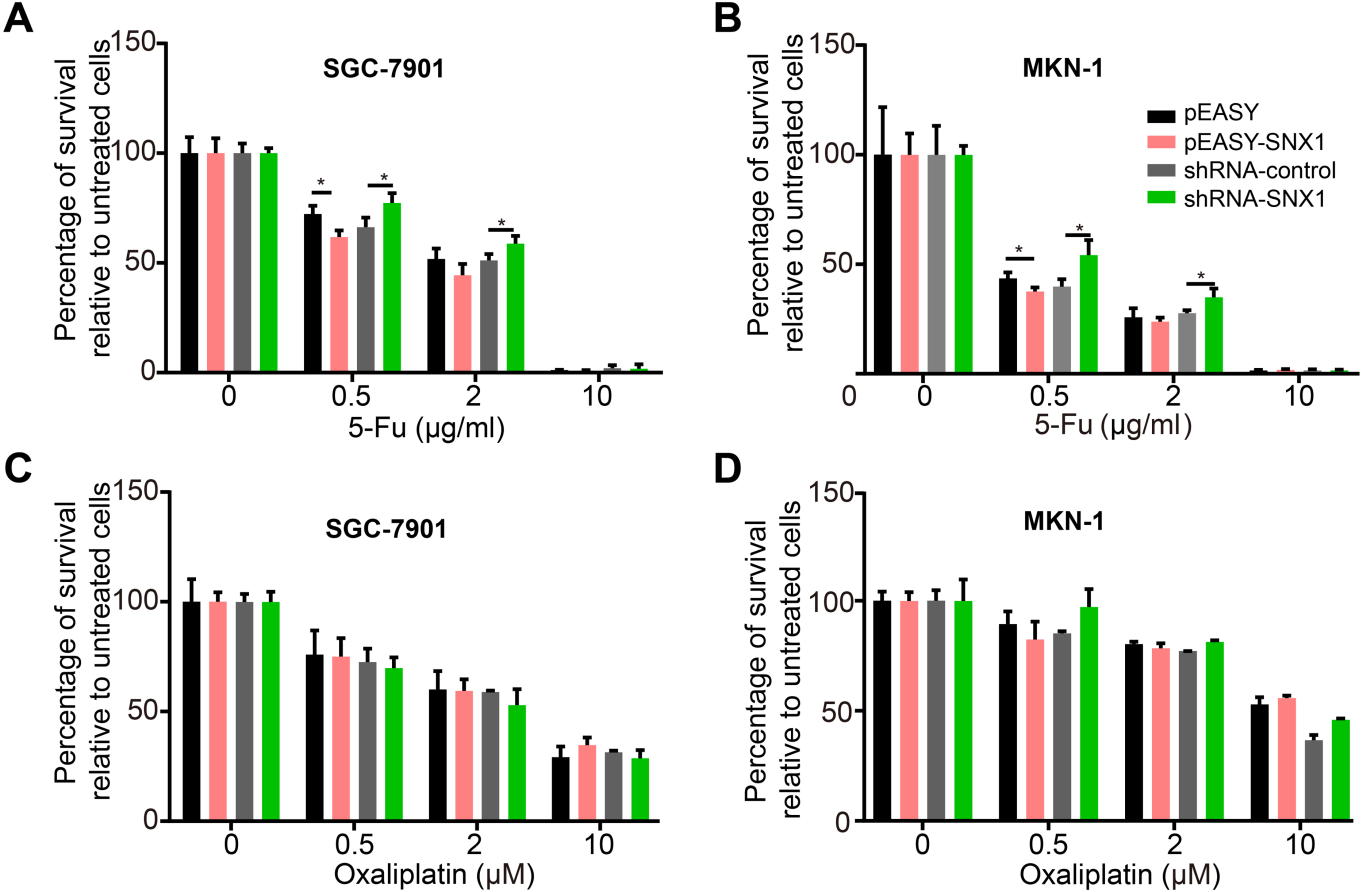
SNX1 increases the sensitivity of GC cells to 5-Fu but not to oxaliplatin. (A–B) Knockdown of SNX1 significantly reduced the sensitivity of GC cells to 5-Fu. (C–D) SNX1 did not increase the sensitivity of GC cells to oxaliplatin. The cell vitality was measured by CCK-8 assay. *p < 0.05; **p < 0.01; ***p < 0.001.

Cell proliferation assay revealed that overexpression of SNX1 inhibited cell proliferation, while knockdown of SNX1 promoted cell proliferation (Fig. 5). A flow cytometry assay was used to detect cell apoptosis. Because annexin V-FITC+/ annexin V-FITC- cell grouping was not obvious, we determined the apoptotic levels of cells by measuring both apoptotic rates (percentage of annexin V-FITC+ cells) and mean apoptotic fluorescence intensity (MFI, mean fluorescence intensity of FITC). It generally showed that cell apoptotic rates and MFI were both lower in GC cells with downregulated SNX1 and higher in GC cells with overexpressed SNX1, indicating that overexpression of SNX1 promoted cell apoptosis although sometimes not significantly (for MKN-1 cells), while knockdown of SNX1 significantly inhibited cell apoptosis (Fig. 6). We also found that overexpression of SNX1 in MKN-1 cells may not always lead to significantly high-level of apoptosis when compared with control. This may be because of the relative higher background expression level of SNX1 in this cell line (Fig. 3), which might lead to the low sensitivity of MKN-1 cells to overexpression of SNX1. Furthermore, we assessed the effect of SNX1 on the response of GC cells to chemotherapy drug 5-Fu and oxaliplatin. As shown in Figs. 7A and  7B, overexpression of SNX1 reduced the survival of cells treated with 5-Fu relative to untreated cells, while knockdown of SNX1 enhanced the cell survival. However, we didn’t find SNX1 upregulated chemo-sensitivity of GC cells to oxaliplatin although SNX1 could enhance apoptosis when cells were treated with oxaliplatin (Figs. 6D– 6H,  7C and  7D). These results together indicated that SNX1 functioned as a tumor suppressor in GC and increased chemo-sensitivity of GC cells to 5-Fu.

### Effects of SNX1 on epithelial-mesenchymal transition (EMT) of GC cells

**Figure 8:**
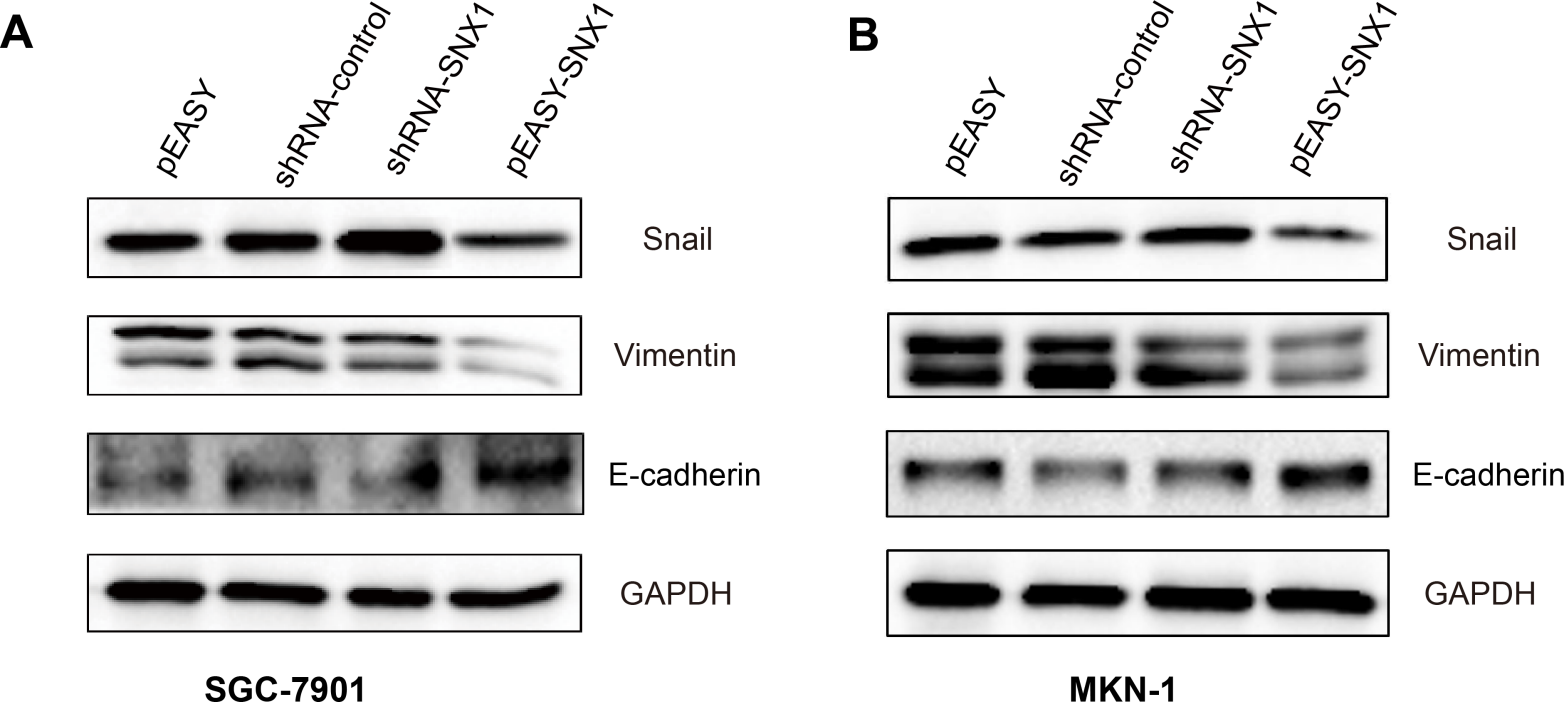
SNX1 regulates the expression of EMT markers. Overexpression of SNX1 leads to the downregulation of Snail and Vimentin and upregulation of E-cadherin in (A) SGC-9701 cells and (B) MKN-1 cells.

We found in the 90 patient with GC that SNX1 was associated with lymph node metastasis (*p* = 0.0286, Table 1). We also found that SNX1 inhibited the migration and invasion abilities of GC cells *in vitro* (Figs. 4D–4L). Thus, we postulated that SNX1 may affect the EMT of GC cells. To test this hypothesis, we investigated the expression of molecular markers epithelial-mesenchymal transition in the cells using WB. Our study suggested that overexpression of SNX1 in GC cells led to downregulation of Vimentin and Snail; and upregulation of E-cadherin (Fig. 8). This might lead to the inhibition of migration and invasion of these cells.

## Discussion

GC remains to be one of the most threatening malignant tumors worldwide. It is a solid tumor with complex genetic and environmental interactions that contribute to its initiation and progression (McLean & El-Omar, 2014). Uncovering the etiology of GC is important for its prevention and treatment, but more detailed mechanisms of the development and progression of GC are still required to research.

As an important member of sorting nexin family, the expression and the potential role of SNX1 in malignancies have rarely been documented yet. The TCGA database favored our research and showed differential expression of SNX1 mRNA in NCTs and GC tissues (Fig. S1). This result led us to further investigate SNX1 protein levels in NCTs and GC tissues. Our histology research strongly suggests that downregulation of SNX1 is a significant factor in the development of human GC. We found that 83.33% (55/60) of the tumor tissues showed immunohistochemical evidence of significant SNX1 reduction (Fig. 1). In addition, study on the 90 patients with detailed follow-up data showed that downregulation of SNX1 in GC was significantly associated with bigger tumor size (*p* = 0.0294), poor tumor stage (*p* = 0.0285) and poor nodal status (*p* = 0.0286) (Table 1), and SNX1 might act as a potential prognostic marker of GC based on the survival curve analysis (Fig. 2). Cox Regression analysis showed that low-level SNX1 was correlated with decreased overall survival of GC patients, and it was an independent risk factor for survival (Table 2). We also found multiple alterations in human GC cells when depletion of this protein or overexpression of this protein (Figs. 4– 8). Knockdown of SNX1 resulted in more colony formation, higher cell migration and invasion abilities (Fig. 4). In addition, downregulation of SNX1 increased proliferation, decreased apoptosis (Figs. 5 and  6). These changes *in vivo* synergize to increase tumor growth and may promote the ability of these tumor cells to metastasize. Downregulation of SNX1 was first reported in human colon cancer (Holdren, Her & Parks, 2005). After that, Nguyen et al. (2006) reported that SNX1 downregulation promoted colon tumorigenesis; increased EGFR phosphorylation and ERK 1/2 signaling. We estimated that the same signaling pathway might also happen in GC cells, and thus increased proliferation with decreased apoptosis following downregulation of SNX1 (Deschenes-Simard et al., 2014). Downregulation of SNX1 by microRNA-95 was reported to induce proliferation in non-small cell lung cancer (Chen et al., 2014). Based on these previous investigations and our present study, we hypothesize that SNX1 may act as an inhibitor of cell proliferation in many tumor cells.

Chemotherapy is an important care for gastric cancer (Fujitani et al., 2016). 5-Fu and oxaliplatin are two of the most effective anticancer regimens in the chemotherapy against numerous solid tumors (Graham, Mushin & Kirkpatrick, 2004; Longley, Harkin & Johnston, 2003). They are also the two common regimens for the treatment of patients with gastric cancer (Inadomi et al., 2017; Li et al., 2010). In this study, we found that knockdown of SNX1 promoted cell proliferation, inhibited cell apoptosis in 5-Fu and oxaliplatin treated cells (Fig. 6), and significantly enhanced the cell survival in 5-Fu treated cells which meant inhibition of chemo-sensitivity of cells to 5-Fu (Figs. 7A and  7B). SNX1 were shown to induce chemoresistance in non-small cell lung cancer (Chen et al., 2014). Bian et al. found histologic evidence that SNX1 expression is downregulated in human colorectal cancer and reported that overexpression of SNX1 enhanced chemo-sensitivity of colorectal cancer cells to both 5-Fu and oxaliplatin (Bian et al., 2016). However, we only found in the GC cells that overexpression of SNX1 enhanced chemo-sensitivity of GC cells to 5-Fu, but not to oxaliplatin. This might be related to different anti-tumor mechanism between the oxaliplatin and 5-Fu and different cell types (Eriguchi et al., 2003; Inada et al., 1997).

As an important protein family, SNXs have 33 members (SNX1 to SNX33). Many of which are involved in intracellular signaling pathway such as Wnt, EGFR, TGF- *β*, Notch etc. (Cullen & Korswagen, 2011; Kurten, Cadena & Gill, 1996; Parks et al., 2001). All of these pathways were reported to be associated with EMT in cancer cells (Holz et al., 2011; Yang et al., 2017; Zhang, Tian & Xing, 2016; Zhang et al., 2017). Thus, SNXs may regulate EMT. SNX27 was recently found to be associated with EMT in breast cancer cells (Li et al., 2015). Our study also indicated that SNX1 was associated with EMT in gastric cancer cells as it showed that overexpression of SNX1 in GC cells inhibited migration and invasion, and led to upregulation of E-cadherin and downregulation of Vimentin and Snail (Fig. 8). David et al. have reported that SNX1 is required for efficient recycling of internalized E-cadherin and re-establishment of epithelial adhesion (Bryant et al., 2007). This may be a potential explanation for SNX1 to regulate EMT, but the exact mechanism needs to be further investigated. EMT played important roles in GC initiation and progression (Katoh, 2005; Peng et al., 2014). The markers of EMT (Vimentin, Snail, and E-Cadherin) were also shown to have special clinical significances to GC. Expression of Vimentin was reported to be associated with decreased survival in GC (Otsuki et al., 2011). Overexpression of Snail was shown to be associated with lymph node metastasis and poor prognosis in patients with GC (Ri et al., 2012). It was also shown to be an independent prognostic predictor for progression and patient survival of GC (He et al., 2012). In this study, we also found that low-level SNX1 was associated with lymph node metastasis, and patients with high-level SNX1 maintained a higher survival rate (Fig. 2, Table 1). These results suggested that SNX1 might regulate GC cell function in a variety of ways.

## Conclusions

In summary, our histology research showed that SNX1 was downregulated in GC tissues and associated with a poor prognosis. This was the first histologic evidence showing SNX1 differential expression between GC and NCTs. We also demonstrated the clinicopathological significance of SNX1 protein expression in GC. Our cytology study on GC cells showed that SNX1 inhibited the colony formation, migration, invasion and proliferation of GC cells, and promoted the cell apoptosis and enhanced the sensitivity of GC cells to 5-Fu. Further study indicated that SNX1 might inhibit the EMT of GC cells. These results together suggested that SNX1 acted as a potential tumor suppressor and prognostic marker in GC, and might serve as a new therapeutic strategy for GC.

##  Supplemental Information

10.7717/peerj.4829/supp-1 Figure S1 Different mRNA levels of SNX1 in GC tissues and NCTs based on the analysis of TCGA database ****(A) Relative mRNA level of SNX1 in 384 GC tissues and 37 NCTs. (B) Relative mRNA level of SNX1 in 34 paired GC tissues and NCTs.

10.7717/peerj.4829/supp-2 Figure S2 Transfection of exogenous SNX1 overexpression plasmid or shRNA plasmid could upregulate or downregulate the SNX1 levels in the GC cells (A, D) Expression of red fluorescence protein (RFP) in GC cells transfected with shRNA-control and shRNA-SNX1 plasmid (derived from the pGPU6-RFP-Neo plasmid) indicates the success of transfection. SNX1 (B–C) SNX1 levels (mRNA and protein) in the MKN-1 cells are upregulated or downregulated by transfection of exogenous SNX1 overexpression plasmid or shRNA plasmid. (E–F) SNX1 levels (mRNA and protein) in the SGC-7901 cells are upregulated or downregulated by transfection of exogenous SNX1 overexpression plasmid or shRNA plasmid. Scale bar (A, D) 100 µm.

10.7717/peerj.4829/supp-3 Table S1 SNX1 mRNA expression levels in 384 GC tissues and 37 non-cancerous paracancerous tissues

10.7717/peerj.4829/supp-4 Table S2 Patients’ information

10.7717/peerj.4829/supp-5 Table S3 Antibodies used in this study

10.7717/peerj.4829/supp-6 Data S1 Raw data

## Additional Information and Declarations

### Data Availability

The following information was supplied regarding data availability: The raw data were uploaded as Supplemental File.
