# Supplementary figures and images for: Sorting nexin-1 is a candidate tumor suppressor and potential prognostic marker in gastric cancer
Source: PeerJ. 2018 May 29;6:e4829. doi: 10.7717/peerj.4829 (PMC5983015; doi:10.7717/peerj.4829)

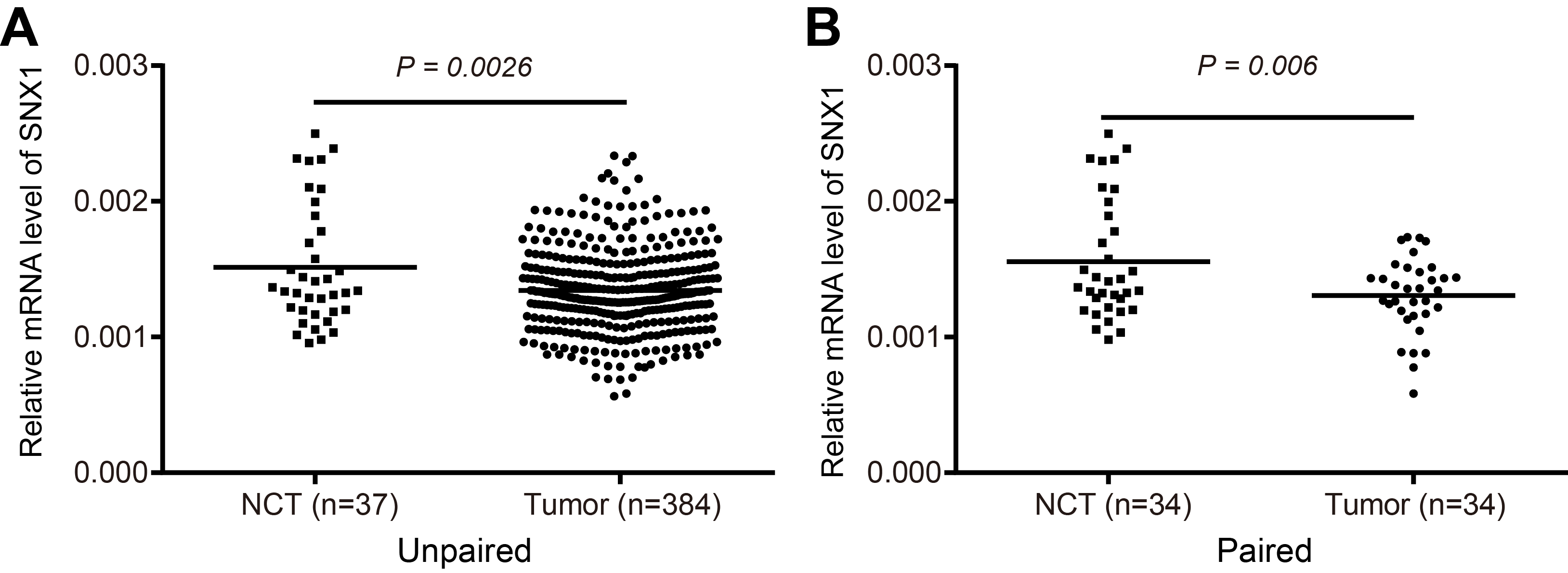

Supplement: Figure S1 — (A) Relative mRNA level of SNX1 in 384 GC tissues and 37 NCTs. (B) Relative mRNA level of SNX1 in 34 paired GC tissues and NCTs. [file peerj-06-4829-s001.png]

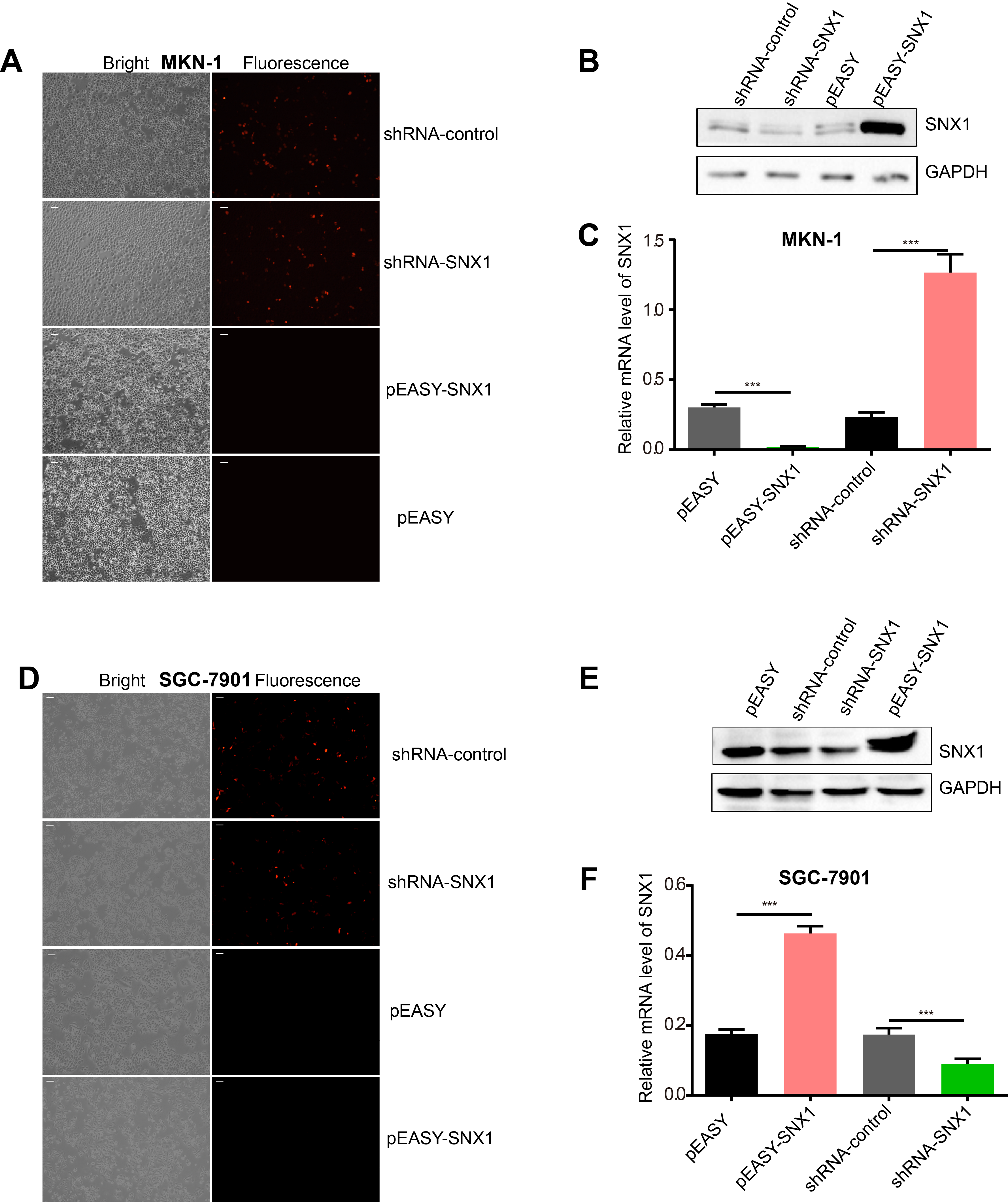

Supplement: Figure S2 — (A, D) Expression of red fluorescence protein (RFP) in GC cells transfected with shRNA-control and shRNA-SNX1 plasmid (derived from the pGPU6-RFP-Neo plasmid) indicates the success of transfection. SNX1 (B–C) SNX1 levels (mRNA and protein) in the MKN-1 cells are upregulated or downregulated by transfection of exogenous SNX1 overexpression plasmid or shRNA plasmid. (E–F) SNX1 levels (mRNA and protein) in the SGC-7901 cells are upregulated or downregulated by transfection of exogenous SNX1 overexpression plasmid or shRNA plasmid. Scale bar (A, D) 100 µm. [file peerj-06-4829-s002.png]

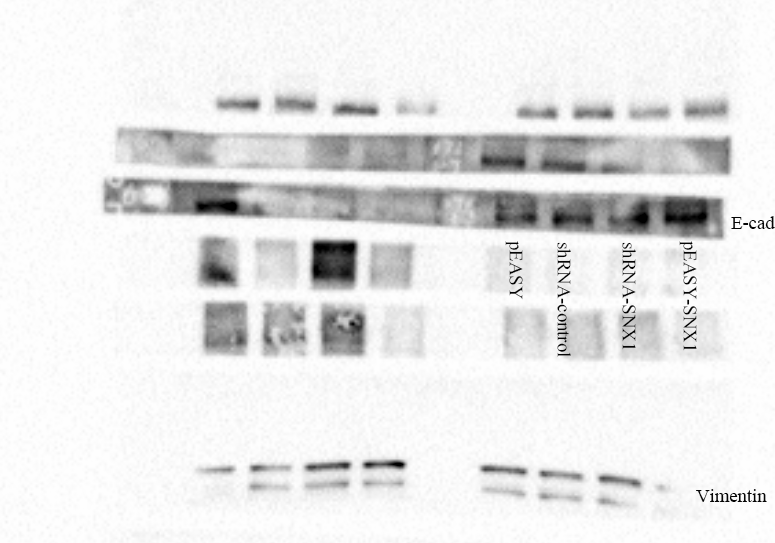

Supplement: Data S1 [file peerj-06-4829-s006.zip › Raw data/original fig/E-Cadherin.tif]

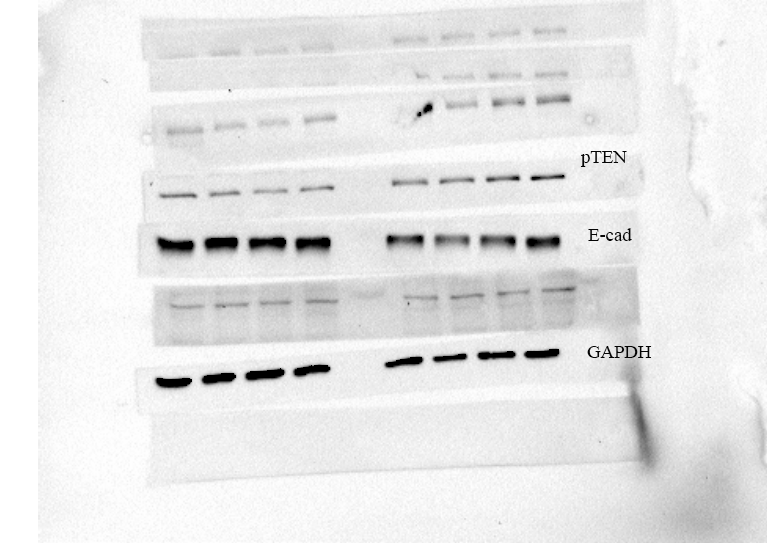

Supplement: Data S1 [file peerj-06-4829-s006.zip › Raw data/original fig/E-cadherin2.tif]

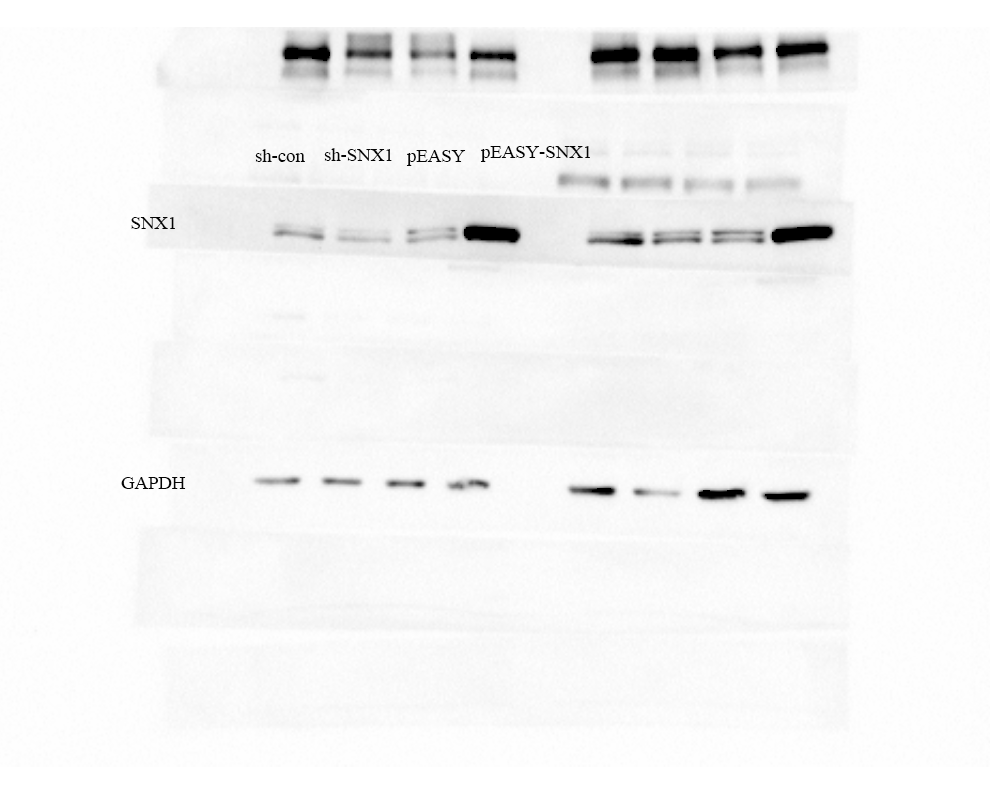

Supplement: Data S1 [file peerj-06-4829-s006.zip › Raw data/original fig/expression was regulated by shRNA or exo1-2.tif]

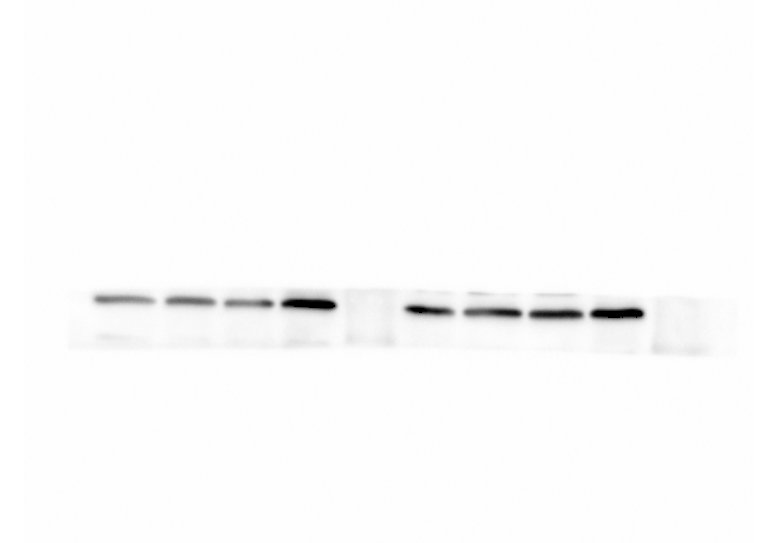

Supplement: Data S1 [file peerj-06-4829-s006.zip › Raw data/original fig/GAPDH for vimentin and e-cad.tif]

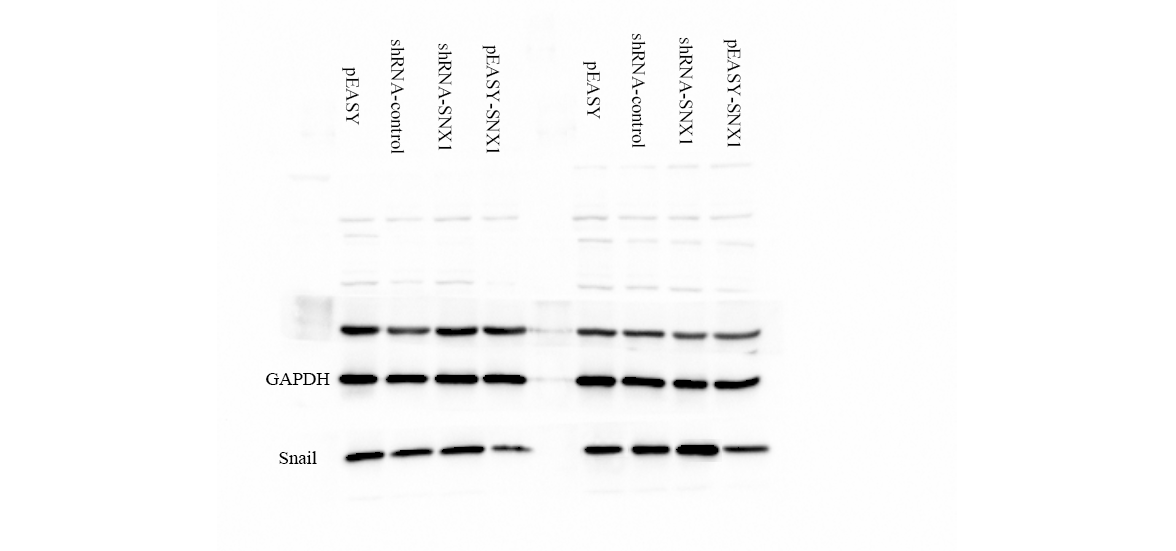

Supplement: Data S1 [file peerj-06-4829-s006.zip › Raw data/original fig/snail1.tif]

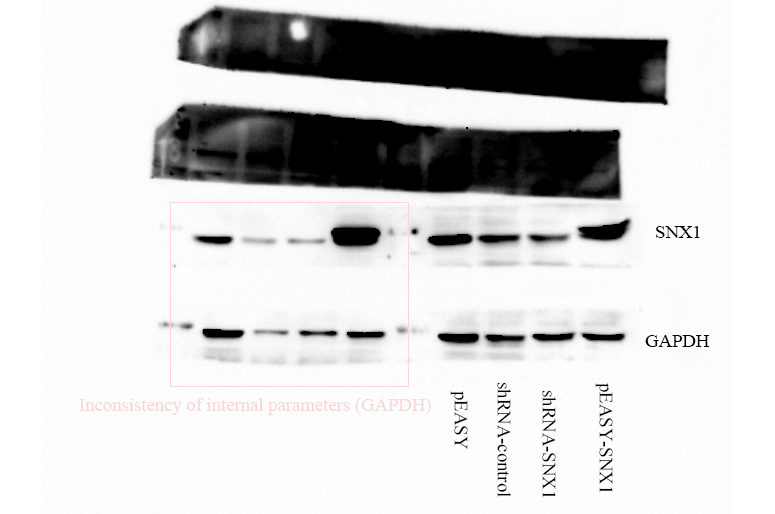

Supplement: Data S1 [file peerj-06-4829-s006.zip › Raw data/original fig/SNX1 expression was regulated by shRNA or exo1.tif]

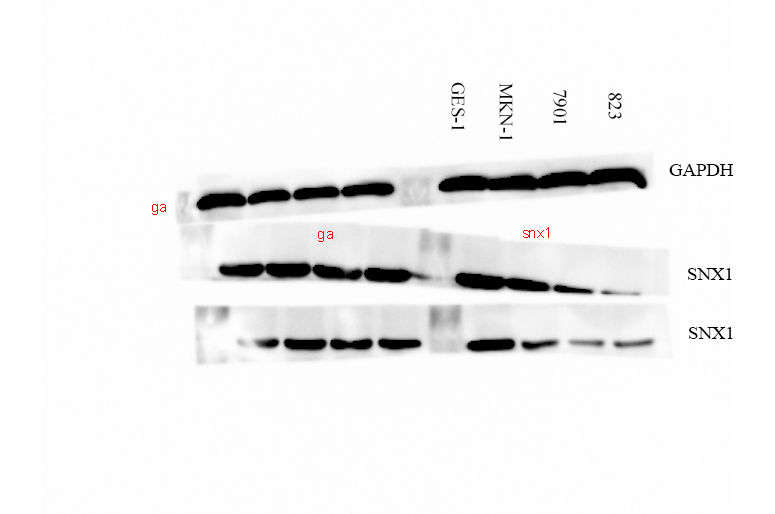

Supplement: Data S1 [file peerj-06-4829-s006.zip › Raw data/original fig/SNX1 in different cells.tif]

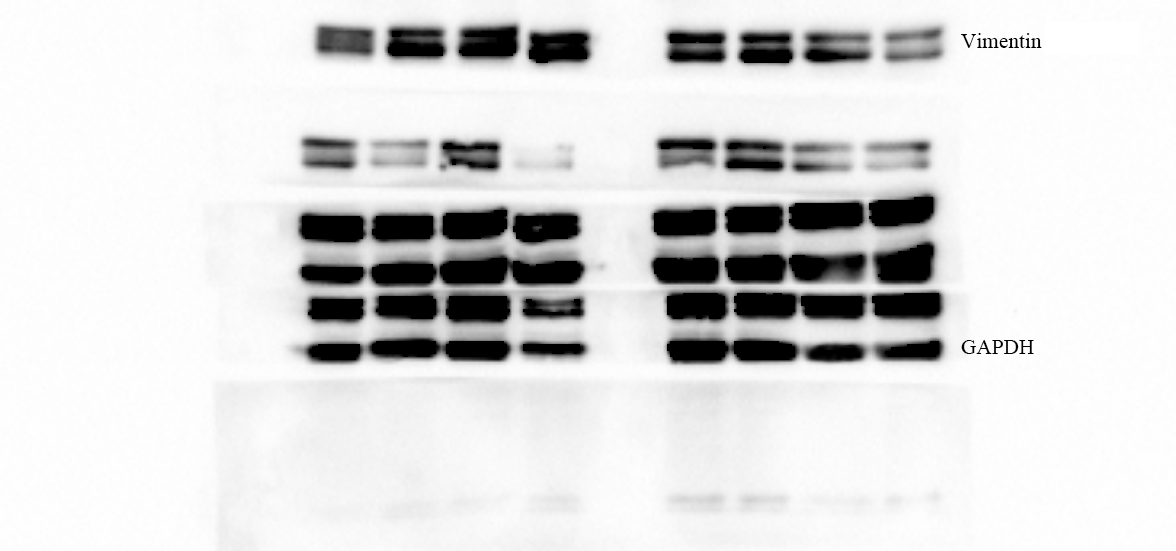

Supplement: Data S1 [file peerj-06-4829-s006.zip › Raw data/original fig/Vimentin 2.tif]

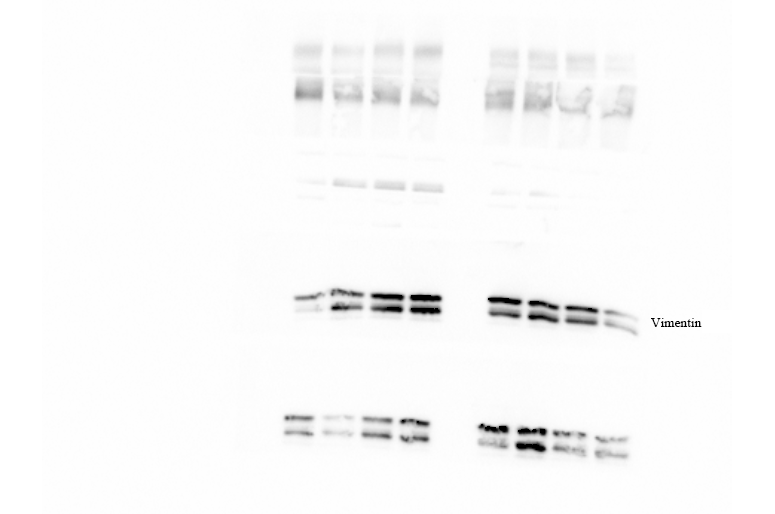

Supplement: Data S1 [file peerj-06-4829-s006.zip › Raw data/original fig/Vimentin.tif]
