# Supplementary material for: Sorting nexin-1 is a candidate tumor suppressor and potential prognostic marker in gastric cancer
Source: PeerJ. 2018 May 29;6:e4829. doi: 10.7717/peerj.4829 (PMC5983015; doi:10.7717/peerj.4829)
Supplement: Table S2 [file peerj-06-4829-s004.doc]

**Table S2. Patients’ information.**

| **Characteristics** | **GC (n=60)^a^** | **GC (n=90)^b^** | Median survival months of the 90 patients | Mean survival months of the 90 patients (Mean±SEM) |
| --- | --- | --- | --- | --- |
| **Age at diagnosis** |  |  |  |  |
| <60 | 24 | 59 | 35.00 | 44.97 ± 4.51 |
| ≧60 | 36 | 31 | 29.00 | 37.75 ± 5.58 |
| **Gender** |  |  |  |  |
| Male | 39 | 54 | 29.00 | 39.04 ± 4.48 |
| Female | 21 | 36 | 41.50 | 47.44 ± 5.66 |
| **Tumor size (cm)** |  |  |  |  |
| ≦ 5 | N/A | 55 | 33.00 | 44.60 ± 4.48 |
| >5 | N/A | 35 | 35.00 | 38.94 ± 4.96 |
| **Regional lymph node** |  |  |  |  |
| N0 | 12 | 20 | 28.50 | 36.55 ± 6.63 |
| N1 | 10 | 30 | 32.50 | 41.70 ± 6.39 |
| N2 | 12 | 24 | 39.00 | 46.63 ± 7.37 |
| N3 | 26 | 16 | 39.50 | 44.69 ± 8.09 |
| **Differentiation** |  |  |  |  |
| G1 | 0 | 0 | - | - |
| G2 | 16 | 22 | 28.50 | 30.00 ± 4.53 |
| G3 | 44 | 68 | 35.00 | 46.41 ± 4.32 |
| **Infiltration degree** |  |  |  |  |
| Tis | 0 | 0 | - | - |
| T1 | 3 | 4 | 25.50 | 26.75 ± 6.95 |
| T2 | 2 | 12 | 28.50 | 34.50 ± 7.84 |
| T3 | 0 | 56 | 32.50 | 44.09 ± 4.80 |
| T4 | 55 | 18 | 38.00 | 45.89 ± 7.54 |
| **p-Stage** |  |  |  |  |
| I | 4 | 10 | 19.00 | 34.40 ± 7.52 |
| II | 5 | 31 | 20.00 | 41.81 ± 6.61 |
| III | 50 | 41 | 85.00 | 43.02 ± 5.19 |
| IV | 1 | 8 | 65.50 | 51.50 ± 11.86 |

*a* Tumor and paracanerous tissues were included in these patients, they were used to study the different expression patterns of SNX1 between tumor tissues and paracancerous tissues.

*b* Tumor tissues and follow-up data were included in these patients, they were used for correlation of SNX1 with clinicopathologic characteristics and for the survival analysis.
