# Supplementary material for: Sorting nexin-1 is a candidate tumor suppressor and potential prognostic marker in gastric cancer
Source: PeerJ. 2018 May 29;6:e4829. doi: 10.7717/peerj.4829 (PMC5983015; doi:10.7717/peerj.4829)
Supplement: Table S3 [file peerj-06-4829-s005.doc]

Table S3. Antibodies used in this study.

| Antibody | Antibody properties | Manufacturer | Catalog number |
| --- | --- | --- | --- |
| SNX1 | Rabbit mAb* | abcam | ab134126 |
| GAPDH | Rabbit mAb/Mouse mAb | CST/Transgen | 5174/HC301 |
| Vimentin | Rabbit mAb | CST | 5741 |
| Snail | Rabbit mAb | CST | 3879 |
| E-Cadherin | Rabbit mAb | CST | 3195 |

* mAb indicates monoclonal antibody
